# Theoretical Study of SO_2_ Selective Detection from the Cr-Modified B_12_N_12_

**DOI:** 10.1021/acsomega.5c02977

**Published:** 2025-06-24

**Authors:** Natanael de Sousa Sousa, Adilson Luís Pereira Silva, Jaldyr de Jesus Gomes Varela Júnior, Nailton Martins Rodrigues

**Affiliations:** † Universidade Federal do Maranhão, 65080-805 São Luís, MA, Brazil; ‡ Universidade Estadual do Maranhão, 65055-310 São Luís, MA, Brazil

## Abstract

Sulfur dioxide is a toxic gas with serious environmental and health implications, making the development of selective and efficient sensors an urgent need. In this work, we investigate, using density functional theory (DFT)/B3LYP-D3/6–31G­(d,p) calculations, the potential of B_12_N_12_ nanocages functionalized with Cr in different configurations (doped, decorated, and encapsulated) for application in SO_2_ chemical sensing. The results show that the encapsulated configuration (Cr@B_12_N_12_) exhibits the best combination of properties, including high electronic sensitivity (Δ*E*_gap_ = 79.3%), moderate adsorption energy (*E*_ads_ = −0.96 eV), and an appropriate recovery time (τ = 167 s), key parameters for reusable sensors under atmospheric conditions. In addition, the system demonstrates high selectivity toward interfering gases such as CO, CO_2_, COCl_2_, CH_4_, H_2_S, N_2_, and H_2_O, corroborated by molecular dynamics simulations. The data analysis suggests that Cr functionalization represents a promising strategy for SO_2_ sensor design, although the system’s performance remains dependent on the adsorption energy range and the experimental feasibility of metal encapsulation.

## Graphical abstract

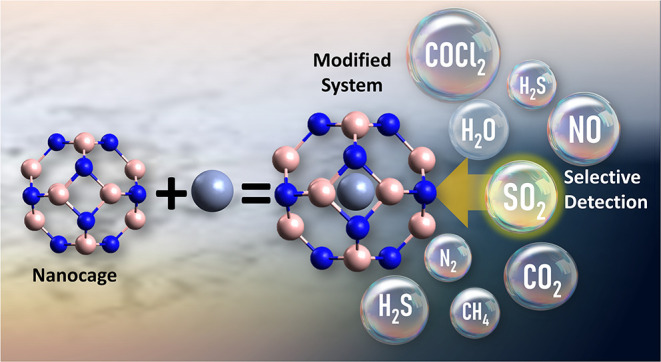

## 1 Introduction

In the context of modern industries, air pollution has become a severe threat to the environment and human health. Among the polluting gases, we can mention SO_2_, a colorless, poisonous, and hazardous gas that poses a significant risk to human health.^1–3^ Obeso et al.^4^ highlighted that developing technologies capable of precisely monitoring specific air pollutants in diverse settings is essential to control emissions and ensure safe exposure limits are not exceeded. Detection and control of gases in residential and industrial environments are necessary, and the study and development of sensors for detecting toxic gases can prevent potentially serious health problems.^5^

In recent decades, theoretical and experimental studies have been carried out to promote the development of new gas sensors and nanoscale materials for removing toxic gases from the environment.^6–8^ Nanomaterials have aroused interest among research groups, motivated by their potential for rapid detection, high sensitivity, and low recovery time.^9–13^ In particular, the B_12_N_12_ nanocage modified with metal atoms has received special attention for improving the sensitivity, adsorption capacity, and selectivity.^8,14–18^

The interaction of SO_2_ gas with various surfaces has already been investigated in theoretical studies using density functional theory (DFT).^19–32^ Soltani et al.^19^ tested the doping of Al_12_N_12_ with Ga and Mg and observed that although the Mg-doped Al_12_N_12_ cluster exhibits high sensitivity to the presence of SO_2_ and NO_2_ molecules, nickel-decorated B_12_N_12_ ^20^ were shown to differentiate SO_2_ from O_3_, and the B_24_N_24_ nanocage,^21^ which differentiates SO_2_ from CS_2_. Badran et al.^22^ showed that Be_12_O_12_ and Mg_12_O_12_ nanocages can be used to detect and remove H_2_S and SO_2_ gases. In a study using the Zn_24_ cluster, Mohammadi et al.^23^ showed that the material can act as an adsorbent for SO_2_, NO, and NO_2_ gases. Albargi et al.^24^ showed that the Cu_2_Zn_10_O_12_ nanocage is a promising candidate for H_2_S and SO_2_ detecting applications. Hussain et al.^25^ demonstrated that the modification in B_12_P_12_ nanocage, particularly the inclusion of zinc, enhances the SO_2_ adsorptive and sensitivity capacities.

The aza-macrocycle^26^ and g-C_3_N_4_,^27^ both pure and modified, emerged as promising materials for SO_2_ and SO_3_ selective detection. The g-C_3_N_4_ showed a higher sensitivity to SO_2_, underscoring its potential for gas detection. Rad and collaborators tested Pt-decorated graphene^28^ and showed that the system detects SO_2_ and O_3_ gases, with better electronic sensitivity. Shamim and collaborators^29^ tested T-graphene (TG), T-boron nitride (TBN), and their heterostructure (TG-TBN) toward CO, SO_2_, NO, and NO_2_ gas molecules and observed that TBN can be used as a gas sensor for SO_2_ and NO_2_ gases. An et al.^30^ and Parkar et al.^31^ tested Fe-doped and Si-doped carbon nanotubes and showed that both systems could detect and remove SO_2_, although Si-doped carbon nanotubes exhibited greater sensitivity. Ahmed et al.^32^ reported that phosphorus-doped T-graphene nanocapsule is a promising candidate for SO_2_ and O_3_ detection.

In addition to the studies cited above,^19–32^ recently, others have been developed using chromium as a surface-modifying element.^33–39^ Hou et al.^33^ tested the Cr_3_-doped GaSe monolayer to detect and remove Cl_2_, NO, and SO_2_ gases and observed that Cr_3_–GaSe monolayer, as a substrate material of disposable resistive sensors and removers (with high recovery times), has enormous potential in the area of detection and removal of these toxic gases. Shao et al.^34^ showed that Cr-doped armchair graphene nanoribbons performed more efficiently than zigzag graphene nanoribbons for SO_2_ detection. Yan et al.^35^ suggested that Cr- and Ti-decorated graphene systems were able to detect SO_2_ and SO_3_. Wu et al.^36^ reported that Cr-NbS_2_ and Mo-NbS_2_ systems could eliminate sulfur-containing gases (SO_2_, H_2_S, and SO_3_) in the atmosphere. Tang et al.^37^ using V-, Cr-, and Mn-decorated MoTe_2_ systems or hazardous gases adsorption observed that Cr-MoTe_2_ and Mn-MoTe_2_ were effective in detecting CH_4_, HCHO, SO_2_, and NO_2_ gases. In this sense, these findings motivated us to design a system by modifying the B_12_N_12_ nanocage with Cr metal, aiming for efficient SO_2_ adsorption and detection in the environment.

Notably, studies on Cr-modified B_12_N_12_ nanocages for SO_2_ adsorption and sensing are absent in the current literature. In the meantime, the objective of this study is to develop a theoretical study at the DFT level to investigate how different B_12_N_12_ nanocage modifications (doped, decorated, and encapsulated) by chromium affect the interaction with SO_2_ and the potential application as a material for SO_2_ selective detection in the environment.

## 2 Computational Methodology

The ground state geometry calculation of isolated B_12_N_12_ and all systems formed by Cr-modified B_12_N_12_ and its interaction with SO_2_ were made using the 6–311G­(d,p) basis set in the ORCA 5.0 program.^40^ The B3LYP functional, which is based on the normalized gradient approximation (GGA), was used because this functional is able to describe pure and modified B_12_N_12_ very well.^19,20^ To improve the description of long-range interaction,^14,15,41,42^ the Grimme dispersion function (B3LYP-D3) was used.^43^ The selected basis set seeks to maintain consistency with previous works^44–46^ and has been extensively used in the literature for studies involving B_12_N_12_ nanocages modified with transition metals,^16,47–50^ showing consistent results and lower computational costs when compared to more elaborate basis sets. Frequency analysis was also performed at the same level of theory to confirm true global minima, and no imaginary frequencies were recorded. The RMS gradient, RMS displacement, maximum gradient, and maximum displacement were 5 × 10^–6^ Hartree, 1 × 10^–4^ Hartree/Bohr, 2 × 10^–3^ Bohr, 3 × 10^–4^ Hartree/Bohr, and 4 × 10^–3^ Bohr, respectively.

The inclusion of Cr metal in B_12_N_12_ was done in five different configurations:^51^ doped (CrB_11_N_12_ and B_12_N_11_Cr, with the replacement of one boron atom and one nitrogen atom by one Cr atom, respectively); decorated (Cr@b_64_ and Cr@b_66_, with a Cr atom positioned above the atomic bond between the tetragonal and hexagonal rings and above the atomic bond between two hexagonal rings, respectively) and encapsulated (Cr@B_12_N_12_, in which a Cr atom is positioned inside the B_12_N_12_ nanocage). The structures were optimized with zero charge and different spin multiplicities, and the lowest energy structures were used in the analyses.

The HOMO–LUMO gap (*E*_gap_) value was calculated for all investigated structures using the equation defined as the energy difference between the frontier molecular orbitals (FMOs).

1 $$E_{\mathrm{gap}}=E_{\mathrm{LUMO}}-E_{\mathrm{HOMO}}$$

where *E*_LUMO_ and *E*_HOMO_ are the energies of the lowest unoccupied molecular orbital and the highest occupied molecular orbital, respectively. The cohesive energy (*E*_coh_ in eq 2) was used to investigate the nanocage’s stability^49,52^

2 $$E_{\mathrm{coh}}=\frac{1}{N}\left(E_{\mathrm{nanocage}}-xE_{B}-yE_{N}-zE_{\mathrm{Cr}}\right)$$

where *E*_nanocage_ is the nanocage total energy (pure or Cr-modified); *E*_B_, *E*_N_, and *E*_Cr_ are the B, N, and Cr atom energies, respectively; *x*, *y*, and *z* are the B, N, and Cr quantities in the structure, respectively, and *N* indicates the total number of atoms.

The ionization potential (IP), electron affinity (eA), chemical hardness (η), and chemical potential (μ) were calculated as a FMOs function, as shown in eqs 3, 4, 5, and 6:^53–55^

3 $$\mathrm{IP}\approx -E_{\mathrm{HOMO}}$$

4 $$\mathrm{eA}\approx -E_{\mathrm{LUMO}}$$

5 $$\eta \approx \frac{1}{2}\left(\mathrm{IP}-\mathrm{eA}\right)$$

6 $$\mu \approx -\frac{1}{2}\left(\mathrm{IP}+\mathrm{eA}\right)$$

A comparison between the stability and reactivity of isolated B_12_N_12_ and the nanocages after interaction with Cr was made since the stability and reactivity of molecular systems can be evaluated using DFT calculations.^56^ Thus, as proposed by Parr et al.,^55^ electrophilicity (ω) can be calculated by the chemical potential and chemical hardness (eq 7)­

7 $$\omega =\frac{\mu^{2}}{2\eta }$$

After modification of the B_12_N_12_ nanocage with Cr, a SO_2_ molecule was adsorbed on the surface of the nanocages and the adsorption energy (*E*_ads_) was calculated using eq 8

8 $$E_{ads}=E_{(nanocage‐SO_{2})}-\left(E_{\left(nanocage\right)}+E_{\left(SO_{2}\right)}\right)+E_{BSSE}$$

where *E*_(nanocage‑SO_2_)_ is the system energy with adsorbed SO_2_, *E*_(nanocage)_ is the energy of the pure or modified nanocage, *E*_(SO_2_)_ is the SO_2_ energy, and *E*_BSSE_ is the basis set superposition error (BSSE).

The charge transfer (*Q*) between the nanocage and SO_2_, which is the charge difference between adsorbed SO_2_ (*Q*_(nanocage‑SO_2_)_) and free SO_2_ (*Q*_(SO_2_)_), was calculated with eq 9.

9 $$Q=Q_{\left(nanocage‐SO_{2}\right)}-Q_{\left(SO_{2}\right)}$$

To aid the adsorption analyses, S–O stretching (*v*_S–O_), Gibbs free energy change (Δ*G*_ads_), distance, and bond order (B.O.) were obtained. Data regarding the density of states (DOS) and molecular electrostatic potential (MEP) were generated with the Multiwfn^57^ and ChimeraX.^58^

To investigate the potential of nanocages as a material for a chemoresistive sensor for SO_2_ detection, the electronic sensitivity of the material to the gas (Δ*E*_gap_), which is related to the electrical conductivity (σ), sensor recovery time (τ), sensitivity (*S*) and selectivity coefficient (κ), and highest occupied molecular orbital-lowest-unoccupied molecular orbital gap (HOMO–LUMO) (*E*_gap_) were calculated. The electrical conductivity (σ) of the sensor is experimentally dependent on the gap energy and can be calculated according to eq 10:^59,60^

10 $$\sigma =AT^{3/2}/e^{-E_{\mathrm{gap}}/2k_{B}T}$$

where *A* (electron/m^3^ K^3/2^) is a constant, *T* is the thermodynamic temperature (*K*), *E*_gap_ is the gap energy, and *k*_B_ is Boltzmann’s constant (8.62 × 10^–5^ eV K^–1^).

After determining the most sensitive nanocage that best adsorbs SO_2_, the recovery time (τ) was calculated using eq 11.^61–63^

11 $$\tau =v_{0}^{-1}e^{-E_{\mathrm{gap}}/k_{B}T}$$

where *v*_0_ are attempt frequencies (1.0 × 10^12^, 5.2 × 10^14^, and 1.0 × 10^16^ s^–1^).^64–66^

The system with the best result for detecting SO_2_ was also subjected to interaction with other gases (CO, COCl_2_, CH_4_, H_2_O, N_2_, CO_2_, H_2_S, and N_2_O), to compare the selectivity of the system for adsorption of SO_2_ with that of other gases. For this, the sensor response (*S*) and selectivity coefficient (κ_*S*O_2_‑int_) were calculated using eqs 12 and 13:^67–69^

12 $$S=\frac{\left|R_{\mathrm{gas}}-R_{\mathrm{pure}}\right|}{R_{\mathrm{pure}}}=\frac{\left|\frac{1}{\sigma_{\mathrm{gas}}}-\frac{1}{\sigma_{\mathrm{pure}}}\right|}{\frac{1}{\sigma_{\mathrm{pure}}}}=\frac{\left|\sigma_{\mathrm{gas}}-\sigma_{\mathrm{pure}}\right|}{\sigma_{\mathrm{gas}}}$$

13 $$\kappa_{SO_{2}‐\mathrm{int}}=\frac{S_{SO_{2}}}{S_{\mathrm{int}}}$$

In eq 12, σ_gas_ represents the conductivity of the gas adsorbed on the nanocage surface, σ_pure_ is the conductance of the isolated nanocage, and *R* is the resistance, which is inversely proportional to the electrical conductivity (see eq 10). In eq 13, *S*_SO_2__ is the SO_2_ sensitivity and *S*_int_ is the sensitivity of other gases.

The SO_2_ sensibility and structural stabilities of the adsorption system were assessed through molecular dynamics (MD) simulations conducted for 500 ps, with a 2 fs integration time step and a dump of 500 fs at room temperature. Interatomic force uses the GFN1 Hamiltonian as implemented in the *x*TB software package.^70^

## 3 Results and Discussion

### 3.1 Spin Multiplicities

Different spin multiplicities of the Cr-modified B_12_N_12_ nanocages (i.e., singlet, triplet, and quintet) were evaluated to determine the most stable configuration of each structure formed. The energy values of the different spin states, referenced to the most stable spin configuration, are presented in Table 1. It is possible to observe that Cr is more stable with a singlet spin state in the encapsulated system (Cr@B_12_N_12_), triplet in the doped systems (CrB_11_N_12_ and B_12_N_11_Cr), and quintet when decorated in B_12_N_12_ (Cr@b_64_ and Cr@b_66_). The formation of Cr-decorated systems is more stable when the metal assumes a high-spin multiplicity; this observation is in agreement with the results published by Arshad et al.^50^

**Table 1:** Relative Energy Values (in Hartree) for Different Cr Spin Multiplicities, Referenced to the Most Stable Cr-Modified B_12_N_12_ System

Cr spin	CrB_11_N_12_	B_12_N_11_Cr	Cr@b_64_	Cr@b_66_	Cr@B_12_N_12_
1	0.036	0.035	0.106	0.114	0.000
3	0.000	0.000	0.044	0.440	0.012
5	0.075	0.030	0.000	0.000	0.024

The spin contamination analysis for Cr-functionalized B_12_N_12_ nanocage revealed varying degrees of spin state purity depending on the metal’s doping or encapsulation position. Table 2 summarizes the obtained values for the total spin operator expectation value, $\left\langle {\hat{S}}^{2}\right\rangle$ , their theoretical expectations, and the corresponding deviations. All systems exhibited deviations below 10%, indicating adequately described spin states with negligible contamination.^71^ This supports the stability of the spin states and the appropriate application of the chosen functional-basis set combination for the studied systems.

**Table 2:** Spin Contamination Analysis of Cr-Modified B_12_N_12_ Nanocages

nanocage	system spin	$\left\langle {\hat{S}}^{2}\right\rangle$	$\left\langle {\hat{S}}^{2}\right\rangle$ expected	deviation
CrB_11_N_12_	4	3.811014	3.750000	0.061014
B_12_N_11_Cr	4	4.097592	3.750000	0.347592
Cr@b_64_	5	6.094094	6.000000	0.094094
Cr@b_66_	5	6.154875	6.000000	0.154875
Cr@B_12_N_12_	1	0.000000	0.000000	0.000000

### 3.2 Structural Analysis

Initially, it was observed that the B_12_N_12_ nanocage ground state geometry is symmetrical and formed by eight hexagonal rings and six tetragonal rings (see Figure 1), with bond lengths of *b*_64_ = 1.484 Å and *b*_66_ = 1.437 Å. This symmetry confers a zero electric dipole moment to B_12_N_12_, and all data are consistent with works from the literature.^72–75^ Furthermore, MEP analysis confirmed that negative charge accumulation occurs around N atoms (red region) and decreases around B atoms (blue region) in the B_12_N_12_ nanocage. For the orbitals, the HOMO is concentrated exclusively on the N atoms, while the LUMO is on the B atoms regions. DOS analysis showed that the pristine B_12_N_12_ nanocage presented an *E*_gap_ = 6.88 eV, which is in good agreement with the results published by Beheshtian et al.^72^ (*E*_gap_ = 6.84 eV) and by Escobedo-Morales et al.^74^ (*E*_gap_ = 6.67 eV).

**Figure 1:**
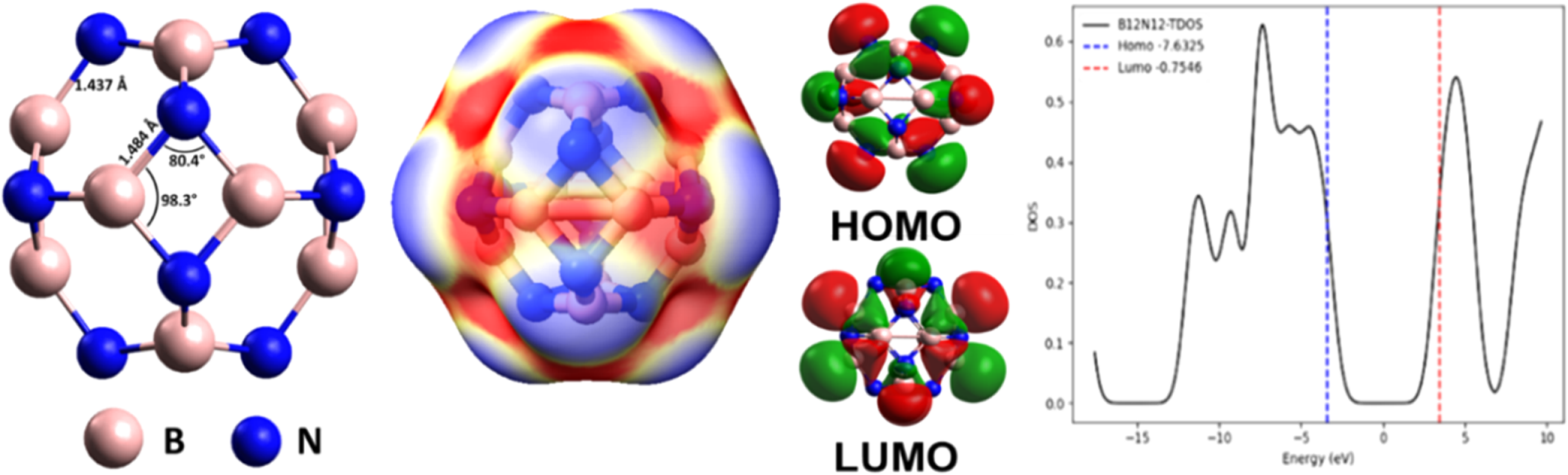
Ground state geometry, MEP, FMOs, and DOS for the B_12_N_12_ nanocage.

The ground-state geometry of the Cr-modified nanocages can be seen in Figure 2. In these systems, the doped structures exhibit local structural deformation caused by the displacement of the Cr atom outward from the nanocage surface; this can be attributed to the larger atomic radius of Cr compared to B and N atoms.^76^ For the decorated Cr@b_66_ and Cr@b_64_ nanocages, it was observed that the B–N bond lengths adjacent to the Cr atom are 0.208 and 0.889 Å longer than those of pure B_12_N_12_, respectively, indicating that decoration also promotes local structural changes. In the encapsulated nanocage (Cr@B_12_N_12_), the B–N bond distances (*b*_66_ = 1.485 Å and *b*_64_ = 1.520 Å) are close to the values found for the unmodified B_12_N_12_ nanocage, indicating that the Cr atom is well accommodated inside the B_12_N_12_ nanocage.^77^

**Figure 2:**
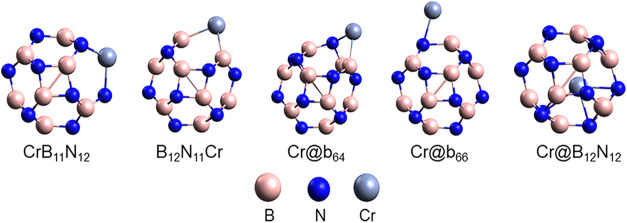
Ground state geometry for B_12_N_12_ nanocages modified with Cr: CrB_11_N_12_, B_12_N_11_Cr, Cr@b_64_, Cr@b_66_, and Cr@B_12_N_12_.

### 3.3 Energy and Stability Properties

Properties of interest of this work for the pure and modified B_12_N_12_ are presented in Table 3, and from this, it is possible to notice a significant increase in the dipole moment after the modification with Cr (0.51–5.58 D); this occurs due to an asymmetry in the nanocage (see Section 3.2). This effect was less pronounced for Cr@B_12_N_12_, and is associated with a little distortion of the nanocages that promotes a smaller separation of charges.

**Table 3:** Dipole Moment (DM) Energies, Ionization Potential (IP), Electron Affinity (eA), Chemical Hardness (η), Chemical Potential (μ), Electrophilicity (ω), Mulliken Charge for Cr Atom (*Q*_Cr_), and Cohesion Energy (*E*_coh_) Values for the Isolated Nanocages

systems	DM/Debye	IP/eV	eA/eV	H/eV	μ/eV	ω/eV	*Q*_Cr_/|e|	*E*_coh_/eV
B_12_N_12_	0.00	7.63	0.75	3.44	–4.19	2.56	-	–7.401
CrB_11_N_12_	3.94	7.14	2.20	2.47	–4.67	4.41	0.81	–7.428
B_12_N_11_Cr	5.17	6.13	2.37	1.88	–4.25	4.79	0.53	–7.180
Cr@b_64_	5.58	5.69	2.10	1.80	–3.89	4.22	0.55	–7.319
Cr@b_66_	5.17	5.47	2.48	1.50	–3.98	5.29	0.46	–7.308
Cr@B_12_N_12_	0.51	4.98	1.46	1.76	–3.22	2.94	–0.53	–7.086

The results showed that the Cr atom exhibits a positive net charge in the doped and decorated structures, suggesting *a* preferential interaction with the regions of highest electron density of the SO_2_ molecule, i.e., with the oxygen atoms. In contrast, in the encapsulated Cr@B_12_N_12_ nanocage, the Cr atom acquires a negative charge, resulting in a lower electron density (i.e., more positive partial charges) on the surrounding boron atoms. This behavior also explains the interaction of the encapsulated nanocage with the negative or partially negative regions of the SO_2_ molecule, as will be further discussed in the next section.

To gain deeper insight into the stability and reactivity of the studied systems, it is necessary to examine the quantum chemical descriptors: chemical hardness (η), chemical potential (μ), and electrophilicity index (ω). The results show that the Cr-modified nanocages present lower η and higher ω values than pristine B_12_N_12_ (see Table 2), indicating that the unmodified nanocage is thermodynamically more stable. According to Parr et al.^55^ and Pearson,^56^ systems with lower η and higher ω values are generally more reactive, supporting the conclusion that Cr-functionalized nanocages exhibit enhanced reactivity compared to the pristine B_12_N_12_. The negative cohesive energy (*E*_coh_) values confirm the thermodynamic feasibility of all studied structures, with CrB_11_N_12_ being the most stable, consistent with previous results,^44^ and, among them, the Cr@B_12_N_12_ nanocage exhibiting notable kinetic stability.^77^

From a practical perspective, experimental studies have demonstrated the feasibility of synthesizing B_12_N_12_ nanocages via top-down approaches, such as plasma discharge and plasma-assisted chemical vapor deposition.^78^ Additionally, the synthesis of BN nanocages modified with encapsulated transition metals such as Fe, Y, Ag, and La has already been achieved, as reported by Oku et al.^79–81^ Therefore, based on the present theoretical results and existing experimental evidence, the synthesis of the Cr@B_12_N_12_ nanocage at both laboratory and industrial scales appears feasible, opening the path for experimental studies involving toxic gases and indicating promising prospects for real-world applications.

### 3.4 SO_2_ Adsorption

In this stage, the adsorption of a single SO_2_ molecule was evaluated, forming the following systems: B_12_N_12_–SO_2_, CrB_11_N_12_–SO_2_, B_12_N_11_Cr–SO_2_, Cr@b_64_–SO_2_, Cr@b_66_–SO_2_, and Cr@B_12_N_12_–SO_2_. The corresponding ground-state geometries are shown in Figure 3. It was observed that the most favorable interaction occurs through the oxygen atom of SO_2_, in agreement with previous studies.^20,33,36^ The adsorption sites exhibiting the strongest interactions are the positively charged Cr atoms in the CrB_11_N_12_, B_12_N_11_Cr, Cr@b_64_, and Cr@b_66_ structures, and the positively charged B atoms in the encapsulated Cr@B_12_N_12_ structure.

**Figure 3:**
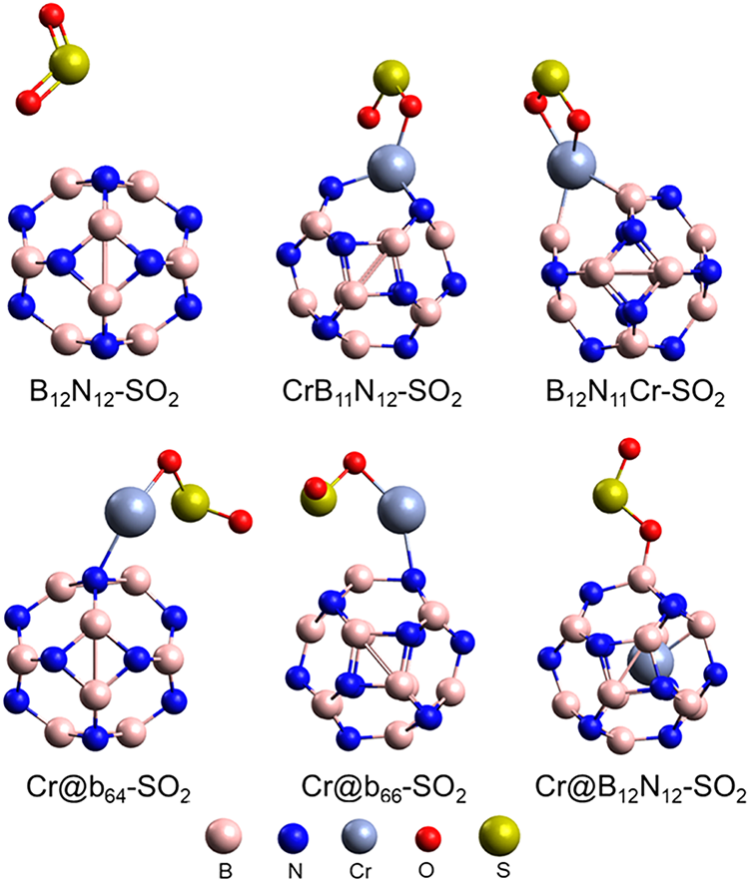
Ground state geometry for the systems: B_12_N_12_–SO_2_, CrB_11_N_12_–SO_2_, B_12_N_11_Cr–SO_2_, Cr@b_64_–SO_2_, Cr@b_66_–SO_2_, and Cr@B_12_N_12_–SO_2_.

For both the isolated and SO_2_-adsorbed systems, the HOMO energy (*E*_H_), LUMO energy (*E*_L_), HOMO–LUMO gap (*E*_gap_), and electronic sensitivity (Δ*E*_gap_) were calculated and are summarized in Table 4. A significant decrease in *E*_gap_ was observed for the Cr-modified systems, with values ranging from 2.69 to 4.95 eV, indicating an increase in the electronic reactivity. This decrease is consistent with previous findings for: (i) B_12_N_12_ doped with Fe, Co, Ni, Cu, and Zn;^18^ (ii) encapsulated 3d, 4d, and 5d transition metals in B_12_N_12_ nanocages;^77^ and (iii) B_12_N_12_ nanocages decorated with first-row transition metals.^82^ Among the Cr-functionalized structures, the Cr@b_66_-decorated nanocage exhibited the most pronounced gap reduction, indicating its elevated reactivity. However, the electronic sensitivity did not correlate directly with this increased reactivity: the pristine B_12_N_12_ nanocage showed a higher sensitivity to SO_2_ adsorption than most Cr-modified systems. Notably, the Cr@B_12_N_12_ nanocage exhibited a sensitivity of 79.3%, highlighting its potential for SO_2_ detection and reinforcing the relevance of its future experimental synthesis, in line with previous transition-metal-based studies^79–81^ and its demonstrated performance in N_2_O detection.^46^

**Table 4:** HOMO Energy (*E*_H_), LUMO Energy (*E*_L_), HOMO-LUMO Gap (*E*_gap_), and Electronic Sensitivity in Percentage (Δ*E*_gap_) Values, for Isolated and SO_2_ Adsorbed Systems for Pure and Modified Nanocages^a^

		isolated			with SO_2_ adsorbed			
system		*E*_H_ (eV)	*E*_L_ (eV)	*E*_gap_ (eV)	*E*_H_ (eV)	*E*_L_ (eV)	*E*_gap_ (eV)	Δ*E*_gap_ (%)
B_12_N_12_		–7.63	–0.75	6.88	–7.50	–4.02	3.48	49.33
CrB_11_N_12_	α	–6.83	–2.79	4.04	–6.59	–3.68	2.91	28.0
	β	–7.14	–2.20	4.95	–7.46	–3.71	3.74	24.3
B_12_N_11_Cr	α	–6.13	–2.37	3.76	–6.44	–2.96	3.48	7.4
	β	–5.56	–2.45	3.12	–6.00	–3.70	2.29	26.5
Cr@b_64_	α	–5.65	–2.38	3.27	–5.93	–2.88	3.05	6.7
	β	–5.69	–2.10	3.60	–5.88	–2.23	3.65	1.4
Cr@b_66_	α	–5.47	–2.48	2.99	–5.89	–2.89	3.00	0.4
	β	–4.86	–2.17	2.69	–5.89	–2.22	3.68	36.8
Cr@B_12_N_12_		–4.98	–1.46	3.53	–4.73	–4.00	0.73	79.3

a α and β indicate spin-up and spin-down, respectively, for the systems that presented an open shell.

Table 5 presents the geometric, spectroscopic, energetic, and electronic parameters used to elucidate the adsorption mechanism of SO_2_ gas on the surfaces of both pristine and chromium-modified B_12_N_12_ nanocages. The equilibrium distances between the SO_2_ molecule and the nanocage surfaces, obtained from ground-state geometries, reveal that SO_2_ remains farther from the pristine B_12_N_12_ nanocage (corroborated by a low bond order <0.1) and significantly closer to the Cr-modified structures, which exhibit markedly higher bond orders ranging from 0.38 to 0.79. These findings indicate that Cr modification enhances the interaction strength between the nanocage and the adsorbed molecule. This conclusion is further supported by the variations in the S–O bond lengths and S–O stretching frequencies observed in the SO_2_-adsorbed complexes (Nanocage–SO_2_) relative to the isolated SO_2_ molecule. For the B_12_N_12_–SO_2_ system, both parameters closely match those of the free SO_2_ molecule, confirming the physisorption character of the interaction.^20^ In contrast, the Cr-modified nanocages exhibit longer S–O bond distances and significant red shifts in the S–O stretching frequencies, indicating substantial orbital overlap and electron density redistribution (clear evidence of chemisorption). It is worth noting that this spectroscopic criterion has previously been employed by our research group to distinguish between physisorption and chemisorption in analogous systems involving modified B_12_N_12_ nanocages interacting with NO,^17^ CNCl,^18^ N_2_O,^46^ and CO.^51^ These precedents reinforce the validity of our current interpretation regarding the nature of the SO_2_ adsorption process.

**Table 5:** Calculated Values of Adsorption Energy (*E*_ads_), Bond Length Cage-SO_2_ (*d*_cage‑SO_2__), Bond Length SO_2_ (*d*_S–O_), Mulliken Charge SO_2_ and Cr (*Q*_SO_2__ and *Q*_Cr_), Dipole Moment (DM), Stretching Frequencies (*v*_S–O_), Cage-SO_2_ Mayer Bond Order (B.O.), and Adsorption Gibbs Free Energy Changes (Δ*G*_ads_) for the Complexes between SO_2_ and Isolated Nanocages

system	*E*_ads_/eV	*d*_cage‑SO_2__/Å	*d*_S–O(1)_/Å	*d*_S–O(2)_/Å	*Q*_SO_2__/|e|	*Q*_Cr_/|e|	DM/Debye	*v*_S–O_/cm^–1^	B.O.	Δ*G*_ads_/eV
B_12_N_12_–SO_2_	–0.19	2.286	1.475	1.461	0.07	-	1.47	1129.48	<0.1	+ 0.20
CrB_11_N_12_–SO_2_	–1.17	1.869	1.618	1.618	–0.31	0.82	1.48	805.10	0.79	–0.91
B_12_N_11_Cr–SO_2_	–1.79	2.024	1.572	1.570	–0.36	0.59	1.98	922.53	0.55	–1.62
Cr@b_64_–SO_2_	–2.03	1.443	1.627	1.485	–0.37	0.67	4.86	814.22	0.70	–1.93
Cr@b_66_–SO_2_	–2.27	1.907	1.612	1.489	–0.37	0.66	5.11	818.68	0.71	–2.18
Cr@B_12_N_12_–SO_2_	–0.96	1.766	1.700	1.492	–0.21	–0.43	3.41	812.48	0.38	–0.63
SO_2_	-	-	1.464	1.464	0.00	-	1.79	1337.49	-	-

The adsorption energy (*E*_ads_) analysis for SO_2_ on the nanocages indicates that the interaction between the SO_2_ molecule and pristine B_12_N_12_ is weak, characterized by a low *E*_ads_ value of 0.21 eV, a bond order below 0.1, and a minimal charge transfer of 0.07|e|.^8,19,20^ These results confirm the physisorption nature of the process, which is further supported by the positive Δ*G*_ads_, indicating a nonspontaneous adsorption under the simulated conditions. In contrast, the introduction of Cr atoms significantly enhanced the adsorption properties of the nanocages. As shown in Table 5, all Cr-modified systems exhibited spontaneous adsorption processes (Δ*G*_ads_ < 0). However, the *E*_ads_ values for the CrB_11_N_12_–SO_2_, B_12_N_11_Cr–SO_2_, Cr@b_64_–SO_2_, and Cr@b_66_–SO_2_ systems were higher than −1.0 eV, ranging between −1.17 and −2.27 eV, thus making their application as chemical sensors unfeasible, as it makes the adsorption process irreversible, with a prolonged adsorption–desorption cycle.^67,83,84^ Notably, these high *E*_ads_ values are confirmed by the high bond orders ranging from 0.55 to 0.79, and by the high charge transfers (greater than −0.31|e|). The *E*_ads_ value of −0.96 eV for the Cr@B_12_N_12_–SO_2_ system, on the other hand, was shown to be adequate since the value is in the range −0.3 eV < *E*_ads_ < −1.0 eV.^84^ This adequacy converges with the moderate values of charge transfer for the SO_2_ molecule (−0.21|e|) and of bond order between the nanocage and the SO_2_ gas (0.38). Furthermore, it is noteworthy that after the interaction with SO_2_, there was an increase in the dipole moment only for Cr@B_12_N_12_, among the modified nanocages, increasing the charge separation sensitivity, which is highly desired for electrochemical sensors.^26^

The interaction between the modified nanocages and the SO_2_ molecule causes changes in *E*_gap_ (see Table 4), as well as in the energy and shape of the frontier molecular orbitals (HOMO and LUMO), as shown in Figure 4, from which it is possible to infer the chemical interaction between the nanocages with Cr and SO_2_. In general, the adsorption of the SO_2_ gas causes a slight stabilization of the HOMO and the LUMO orbitals for the CrB_11_N_12_, B_12_N_11_Cr, Cr@b_64_, and Cr@b_66_ nanocages. On the other hand, for the Cr@B_12_N_12_ nanocage, there was a weak destabilization of the HOMO (0.25 eV) and a significant stabilization of the LUMO (2.54 eV), which resulted in a greater electronic sensitivity for the Cr@B_12_N_12_ nanocage. Furthermore, from the DOS diagrams, it is possible to observe a preponderant role of the cage in the formation of frontier molecular orbitals after interaction with SO_2_. However, a greater participation of chromium in the formation of LUMO was observed for the Cr@B_12_N_12_–SO_2_ system, confirming that the Cr-encapsulated nanocage is more sensitive to the SO_2_ molecule. The MEPs confirm an increase in the electron density distribution in SO_2_ (accumulation of electronic charges), meaning that SO_2_ receives a certain amount of charge from the modified nanocages (negative Mulliken charges).

**Figure 4:**
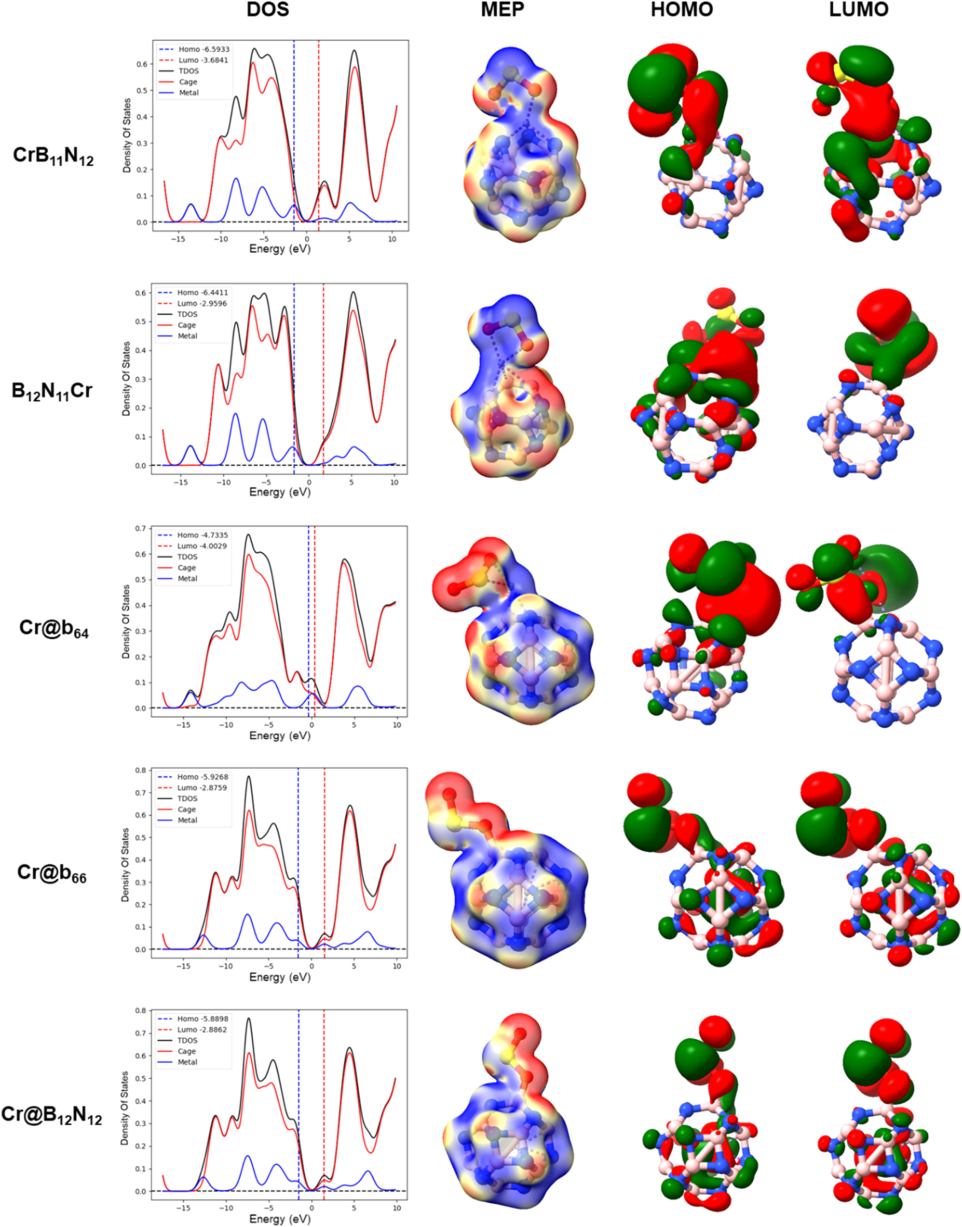
DOS, MEP, and HOMO and LUMO data, for ground state geometry of the CrB_11_N_12_–SO_2_, B_12_N_11_Cr–SO_2_, Cr@b_64_–SO_2_, Cr@b_66_–SO_2_, and Cr@B_12_N_12_–SO_2_ systems.

### 3.5 Recovery Time

The interaction of the SO_2_ molecule on the Cr@B_12_N_12_ nanocage surface showed moderate adsorption energy (−0.96 eV) and high sensitivity to SO_2_ (79.3%); for this reason, the recovery time (τ) for the Cr@B_12_N_12_ nanocage was also evaluated. The value was approximately 167 s, a suitable recovery time for chemical sensor applications.^84^ Different recovery times depending on the temperature (*T*) and the attempt frequency (*v*_0_) can be observed in Table 6,^46,51^ and it is possible to observe that the recovery times calculated for Cr@B_12_N_12_–SO_2_ varied between 0.51 μs and 4.63 h for the attempt frequencies and temperatures tested. Confirming that, at 298.15 K and under visible light, the recovery time of the Cr@B_12_N_12_ nanocage presents a suitable result for applications such as the SO_2_ sensor, it was also possible to observe that the recovery time can be reduced with the use of ultraviolet light or with the increase in temperature.

**Table 6:** Calculated Values of Recovery Time (τ) for SO_2_ Adsorption on Cr@B_12_N_12_ Nanocage Using Three Different Attempt Frequencies and Temperatures

		recovery time		
light	*v*_0_ (s^–1^)	298.15 K	398.15 K	498.15 K
infrared	1.0 × 10^12^	4.63 h	1.41 s	5.12 ms
yellow	5.2 × 10^14^	166.8 s	14 ms	0.05 ms
ultraviolet	1.0 × 10^16^	1.67 s	0.14 ms	0.51 μs

A more comprehensive approach to the recovery time (τ) behavior for the Cr@B_12_N_12_–SO_2_ system as a function of a temperature variation from 250 to 450 K can be observed in Figure 5, considering the evaluated temperatures. It is possible to notice that τ tends to 10 s, for example, at 280 K (ultraviolet light), 325 K (yellow light), and 380 K (infrared light), indicating that it is possible to adjust the desorption process of a molecule on a sensitive surface. When room temperature is available, it is observed that for infrared light, the time for desorption of the SO_2_ molecule is greater than 4 h, which makes it impossible to apply the Cr@B_12_N_12_ nanocage as a sensor for intermittent use in the detection of SO_2_. In this sense, the Cr@B_12_N_12_ nanocage is suitable for application in disposable chemical sensors to rapidly detect the toxic gas SO_2_, taking advantage of the high electronic sensitivity and adequate recovery time using visible light and adsorption energy.

**Figure 5:**
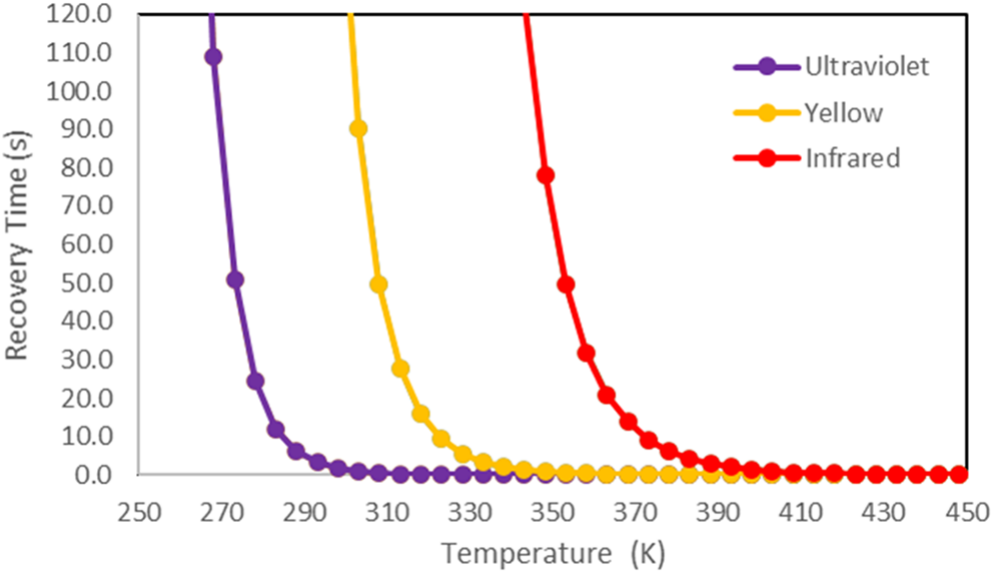
Variation in recovery time of the Cr@B_12_N_12_–SO_2_ adsorption system as a function of the temperature and attempt frequency.

### 3.6 Effect of Interfering Gases

The selectivity of the Cr@B_12_N_12_ nanocage toward SO_2_ was further evaluated in the presence of potential interfering gases, namely, CO, COCl_2_, CH_4_, H_2_O, N_2_, CO_2_, H_2_S, and N_2_O. The optimized geometries of the adsorption complexes are shown in Figure 6.

**Figure 6:**
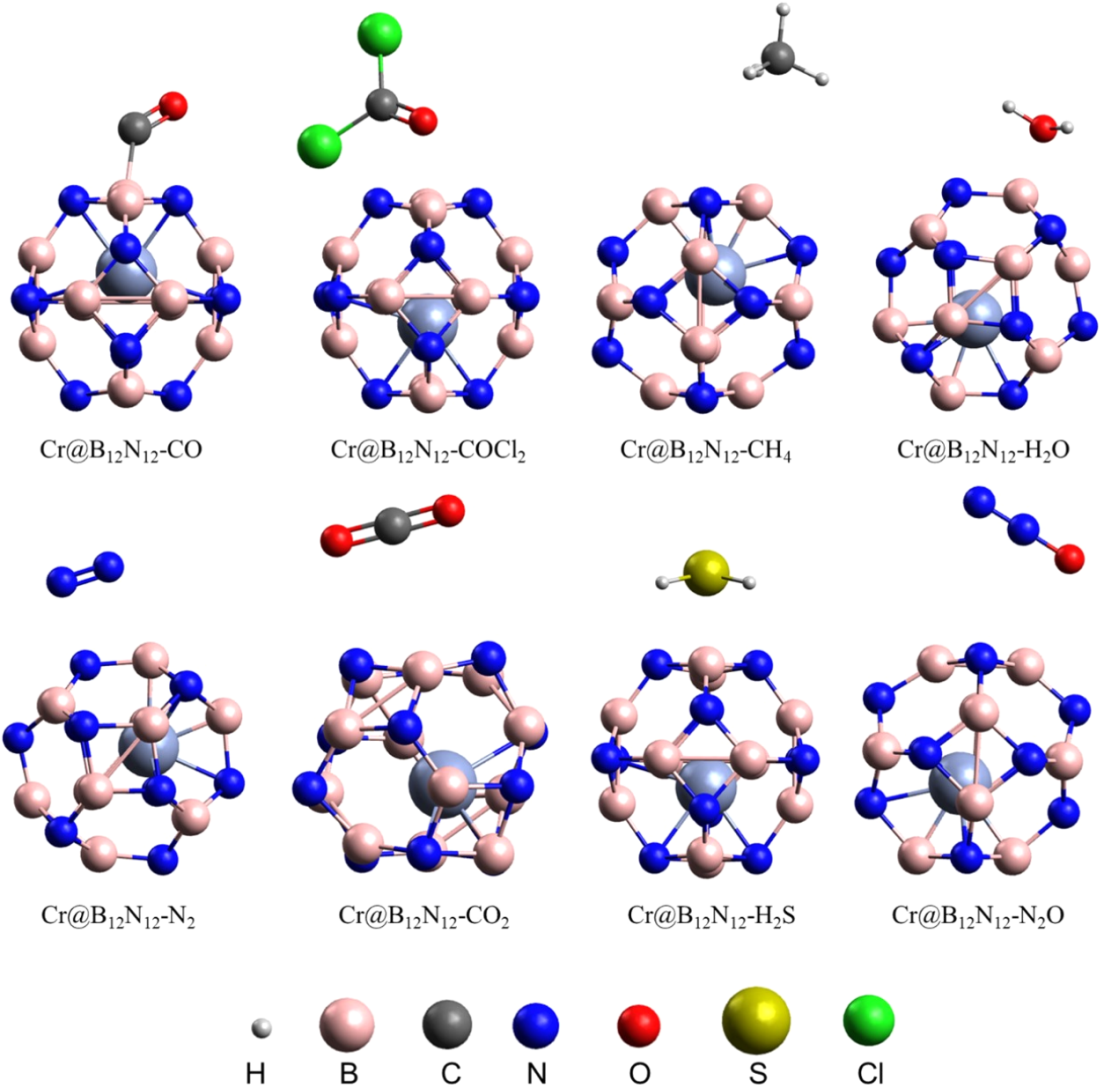
Optimized structures of the Cr@B_12_N_12_ nanocage and the adsorbed gases CO, COCl_2_, CH_4_, H_2_O, N_2_, CO_2_, H_2_S, and N_2_O.

Table 7 summarizes the key parameters associated with the adsorption of each gas on the Cr@B_12_N_12_ nanocage, including adsorption energy (*E*_ads_), HOMO–LUMO energy gap (*E*_gap_), electronic sensitivity (Δ*E*_gap_), sensor response (*S*), selectivity coefficient (κ), and charge transfer to the gas (*Q*_gas_). The data clearly indicate that SO_2_ exhibits the strongest interaction with the nanocage, with the highest electronic sensitivity among all gases studied. Notably, only H_2_O was found to be chemisorbed, whereas CO, COCl_2_, CH_4_, N_2_, CO_2_, H_2_S, and N_2_O showed weak physisorption, with absolute *E*_ads_ values below 0.3 eV, indicating limited interaction with the Cr@B_12_N_12_ surface.

**Table 7:** Values of Adsorption Energy (*E*_ads_), HOMO-LUMO Gap (*E*_gap_), Electronic Sensitivity (Δ*E*_gap_), Sensitivity (*S*), Selectivity Coefficient (κ), and Mulliken Charge SO_2_ (*Q*_SO_2__) Calculated for the Interaction of Gases CO, COCl_2_, CH_4_, H_2_O, N_2_, CO_2_, H_2_S, and N_2_O with Cr@B_12_N_12_

system	*E*_ads_/eV	*E*_gap_/eV	Δ*E*_gap_/%	*S*	κ_SO_2_‑int_	*Q*_SO_2__/|e|
Cr@B_12_N_12_–SO_2_	–0.96	0.73	79.30	4.5 × 10^23^		–0.207
Cr@B_12_N_12_–CO	–0.07	1.97	44.14	1.4 × 10^13^	3.0 × 10^10^	0.070
Cr@B_12_N_12_–COCl_2_	–0.19	2.30	34.96	2.7 × 10^10^	1.7 × 10^13^	0.088
Cr@B_12_N_12_–CH_4_	–0.06	3.60	2.03	0.75	5.9 × 10^23^	0.015
Cr@B_12_N_12_–H_2_O	–0.67	3.47	1.64	2.08	2.1 × 10^23^	0.243
Cr@B_12_N_12_–N_2_	–0.06	3.51	0.57	0.48	9.4 × 10^23^	0.019
Cr@B_12_N_12_–CO_2_	–0.13	3.51	0.49	0.40	1.1 × 10^24^	0.022
Cr@B_12_N_12_–H_2_S	–0.26	3.52	0.24	0.18	2.5 × 10^24^	0.269
Cr@B_12_N_12_–N_2_O	–0.13	3.52	0.14	0.10	4.3 × 10^24^	0.040

Charge transfer analysis revealed that, for the Cr@B_12_N_12_–SO_2_ system, electron flow occurs from the nanocage to the SO_2_ molecule (−0.207|e|), suggesting the presence of back-donation mechanisms in the interaction, in agreement with previous studies.^18,51^ Conversely, for all other interfering gases, the direction of charge transfer was predominantly from the gas molecules to the nanocage.

The sensitivity parameter (*S*) quantifies the change in electronic properties of the nanocage upon gas adsorption, while the selectivity coefficient (κ) reflects the nanocage’s capability to distinguish SO_2_ in a gas mixture.^85^ Higher values of κ indicate a better discrimination capability. The results confirm that the Cr@B_12_N_12_ nanocage exhibits excellent selectivity and sensitivity toward SO_2_ detection, suggesting its applicability in the development of high-performance chemical sensors for atmospheric monitoring.

To investigate the thermodynamic stability of the studied adsorption system and to reinforce the analysis of the nanocage’s selectivity toward SO_2_ in the presence of interfering gases, a more realistic system was subjected to a 500 ps molecular dynamics (MD) simulation. The system consisted of a Cr@B_12_N_12_ nanocage surrounded by SO_2_ gas and all other gases tested. The graph in Figure 7 (left side) shows an abrupt drop in the system’s energy at around 100 ps of the MD simulation, which is related to the dispersion of CH_4_, N_2_, CO_2_, and N_2_O gases, none of which bind to the nanocage. CO, H_2_S, and water molecules bind to the nanocage, whereas COCl_2_ remains near the system but does not interact directly with the cage. In contrast, the SO_2_ molecule binds both to the metal center and to a neighboring boron site via its two oxygen atoms, establishing a stronger interaction with the nanocage throughout the trajectory, as illustrated in the system snapshot at the end of the MD run (Figure 7, right).

**Figure 7:**
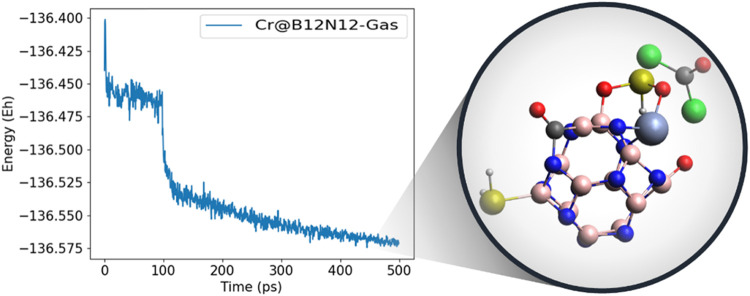
Molecular dynamics analysis of the Cr@B_12_N_12_ nanocage with SO_2_, CO, COCl_2_, CH_4_, N_2_, CO_2_, H_2_S, and N_2_O gases. Trajectory graph (left) and final state of the trajectory (right).

The molecular dynamics analysis showed that interactions with higher sensitivity (such as CO Δ*E*_gap_ = 44.14%) or with higher relative adsorption energy values (such as H_2_O *E*_ads_ = −0.67 eV and H_2_S *E*_ads_ = −0.26 eV) are possible in realistic systems. However, these do not interfere with the detection or adsorption of the SO_2_ gas. The MD results reaffirm the system’s stability and selectivity. The MD results, therefore, corroborate both the thermodynamic stability and selectivity of the Cr@B_12_N_12_ nanocage toward SO_2_, reinforcing its potential as a robust sensing material under practical operational conditions.

Finally, a comparative analysis between the Cr@_12_N_12_ nanocage and other materials reported in the literature for SO_2_ adsorption^19–32^ is presented in Table 8. The Cr@B_12_N_12_ nanocage demonstrated one of the highest electronic sensitivities (Δ*E*_gap_ = 79.3%) among the systems investigated, outperforming most candidates and closely followed by the MgB_11_N_12_ nanocage (Δ*E*_gap_ = 75.37%) and the phosphorus-doped tetragonal graphene nanocapsule (P-doped TGC, Δ*E*_gap_ = 74.19%). Despite its high sensitivity, the P-doped TGC presents a weak interaction with SO_2_ (*E*_ads_ = −0.21 eV), which may impair selectivity in the presence of interfering gases.^51,84^ Conversely, the MgB_11_N_12_ nanocage exhibits excessively strong binding (*E*_ads_ = −3.39 eV), which compromises sensor reusability due to irreversible adsorption. In contrast, the Cr@B_12_N_12_ nanocage achieves an optimal balance between adsorption energy and sensitivity, combining selective SO_2_ detection with feasible desorption and regeneration properties. These findings highlight the Cr@B_12_N_12_ nanocage as a highly promising candidate for the design of efficient, selective, and reusable chemoresistive sensors for SO_2_ monitoring in atmospheric environments.

**Table 8:** Comparison of Sensing Materials for the Detection of Sulfur Dioxide (SO_2_)

sensor	functional/basis set	*E*_ads_/eV	Δ*E*_gap_/%	refs
GaB_11_N_12_	B3LYP/6–31G(d)	–2.80^a^	67.45^c^	Soltani et al.^19^
MgB_11_N_12_	B3LYP/6–31G(d)	–3.39^a^	75.37^c^	Soltani et al.^19^
Ni-decorated B_12_N_12_	B3LYP/6–31G(d,p)	–1.82^a^	36.90^c^	Rad and Ayub^20^
B_24_N_24_	B3LYP-D3/6–31G(d)	–0.49^b^	45.87^d^	Ding and Gu^21^
Be_12_O_12_	B3LYP-D3/6–31G(d,p)	–0.68^b^	15.97^d^	Badran et al.^22^
Mg_12_O_12_	B3LYP-D3/6–31G(d,p)	–2.11^b^	2.60^d^	Badran et al.^22^
Zn_24_	TPSSh/Lanl2DZ	–4.26^a^	46.08^c^	Mohammadi et al.^23^
Zn_12_O_12_	TPSSh/Lanl2DZ	–0.54^a^	55.13^c^	Mohammadi et al.^23^
Cu_2_Zn_10_O_12_	B3LYP/LanL2DZ	–2.64^a^	4.98^d^	Albargi et al.^24^
B_12_P_12_	B3LYP/6–31G(d,p)	–0.15^a^	45.67^c^	Hussain et al.^25^
Zn–B_12_P_12_	B3LYP/6–31G(d,p)	–1.08^a^	55.97^c^	Hussain et al.^25^
aza-macrocycle	ωB97XD/6–31 + G(d,p)	–0.24^b^	1.29^c^	Siddique et al.^26^
g-C_3_N_4_	B3LYP/6–31G(d,p)	–0.28^b^	7.45^c^	Ashiq et al.^27^
Pt-decorated graphene	B3LYP/6–31G(d,p)	–0.85^b^	25.10^c^	Rad and Zareyee^28^
T-boron nitride (TBN)	PBE/DNP	–0.911^a^	6.5^d^	Shamim et al.^29^
Fe-CNT	PBE/DNP	–1.298^a^	28.40^d^	An et al.^30^
Si-CNT	PBE/DNP	–1.66^a^	42.59^c^	Parkar et al.^31^
P-doped TGC	B3LYP-D3/6–31G(d)	–0.21^b^	74.19^c^	Ahmed et al.^32^
Cr@B_12_N_12_	B3LYP-D3/6–31G(d,p)	–0.96	79.30	This work

a Without BSSE correction.

b With BSSE correction.

c Calculated value using *E*_gap_ from the literature.

d Literature data.

## 4 Conclusions

In this theoretical study, pristine and chromium-modified B_12_N_12_ nanocages (doped, decorated, and encapsulated) were evaluated as potential candidates for the selective sulfur dioxide (SO_2_) detection. Among the investigated systems, the Cr@B_12_N_12_ nanocage exhibited the most balanced performance, with moderate adsorption energy (*E*_ads_ = −0.96 eV), high electronic sensitivity (Δ*E*_gap_ = 79.3%), and an adequate recovery time at room temperature (τ = 167 s). These values indicate a favorable balance between effective detection and reversible adsorption, which are key features for reusable gas sensors.

Importantly, Cr@B_12_N_12_ showed remarkable selectivity toward SO_2_ in the presence of common atmospheric interfering gases, including CO, COCl_2_, CH_4_, N_2_O, N_2_, CO_2_, H_2_S, and water, confirmed by molecular dynamics tests. While H_2_O exhibited chemical adsorption, its electronic sensitivity (Δ*E*_gap_ = 1.64%) was significantly lower than that of SO_2_. All other gases showed weak physisorption (*E*_ads_ < 0.3 eV) and minimal gap variation, confirming SO_2_-specific detection.

Taken together, these findings position Cr@B_12_N_12_ as a promising platform for SO_2_ chemoresistive sensing applications. Nevertheless, the adsorption energy is near the upper limit for reversible detection, and the experimental synthesis of encapsulated Cr within the nanocage remains a challenge. Therefore, additional studies, particularly experimental validation and stability assessments, are necessary to support the practical implementation of this system in real-world sensor technologies.

## §

Date of death: June 10, 2024
